# Microstructures and Tensile Properties of 9Cr-F/M Steel at Elevated Temperatures

**DOI:** 10.3390/ma15031248

**Published:** 2022-02-08

**Authors:** Guangjie Zhang, Qinggang Zhang, Junfeng Yang, Zhuoming Xie, Linchao Zhang, Rui Liu, Gang Li, Hui Wang, Qianfeng Fang, Xianping Wang

**Affiliations:** 1 Key Laboratory of Materials Physics, Institute of Solid State Physics, Hefei Institutes of Physical Science, Chinese Academy of Sciences, Hefei 230031, China; gjzhang@mail.ustc.edu.cn (G.Z.); zhangqinggang2016@163.com (Q.Z.); zmxie@issp.ac.cn (Z.X.); lczhang@issp.ac.cn (L.Z.); liurui@issp.ac.cn (R.L.); qffang@issp.ac.cn (Q.F.); 2 Scinece Island Branch, Graduate School of University of Science and Technology of China, Hefei 230026, China; 3 Institutes of Physical Science and Information Technology, Anhui University, Hefei 230601, China; 4 Lu’an Branch, Anhui Institute of Innovation for Industrial Technology, Lu’an 237100, China; 5 Science and Technology on Reactor Fuel and Materials Laboratory, Nuclear Power Institute of China, Chengdu 610041, China; 13880784511@163.com; 6 Interdisciplinary Materials Research Center, Institute for Advanced Study, Chengdu University, Chengdu 610106, China; qinghe5525@163.com

**Keywords:** ferrite-martensite steel, precipitates, tensile properties, microstructure

## Abstract

Tensile properties and microstructure changes under different stress states of tempered 9Cr-F/M steel were characterized using a transmission electron microscope (TEM), electron backscatter diffraction (EBSD), scanning electron microscopy (SEM), Vickers hardness tester, and tensile tester. This tempered steel has a typical lath martensite structure with only a few polygonal ferrites embedded, and M_23_C_6_ and MX phases nucleated on the lath boundaries or within the sub-grains. At elevated temperatures, the strength of the steel decreases. However, the elongation at 400 °C is lower than that at room temperature. For the necking zone, tensile deformation made the grain elongated to the direction of applied stress and thus the grain’s cross-section becomes smaller. For samples with rectangular working area cross-section, the deformation in the TD direction was more severe than that in the ND direction, which made the grain elongated in the TD direction. These results can provide some guidance for composition optimization of the 9Cr-F/M steel and facilitate a better understanding of the fracture mechanism under different stress states.

## 1. Introduction

Nuclear fission energy, as one kind of clean, high energy density, and environmentally friendly energy, is considered as one of the most promising solutions to meet the ever-increasing energy demand [1,2]. Lead-cooled fast reactor (LFR) is one of the six conceptual reactors of the fourth-generation fast reactor (Gen-IV) and represents the frontier of nuclear fission energy development owing to unique characteristics including good neutron economy, good thermal performance, better chemical stability, and large safety margin [3,4]. Compared with light water reactors (LWRs) under service currently, fuel cladding materials (FCMs), which function as both the container of nuclear fuel and the barrier to prevent the leak of radioactive fission products in a nuclear reactor, will suffer much harsher conditions in LFR, such as liquid Pb-Bi eutectic (LBE) coolant corrosion, elevated service temperature, and their combination with neutron irradiation [3,4]. These offer great challenges to engineers and designers on cladding materials selection [5].

Owing to superior thermal conductivity, thermal expansion, resistance to helium radiation-induced swelling and embrittlement, 9–12% Cr-F/M steels have been widely researched [6,7,8]. Among them, the 9Cr-F/M steels developed by the United States and the European Union have excellent high-temperature mechanical properties and radiation swelling resistance and therefore are considered as the most promising fuel cladding materials for LFR [9].

However, for 9Cr-F/M steels, their poor resistance to LBE corrosion and obvious corrosion hardening have already been the major obstacles limiting their practical application as fuel cladding materials for LFR. It has been reported that silicon (Si) could significantly enhance the corrosion resistance of 9Cr-F/M steel to LBE through the formation of a layer of Si-enriched oxide film on its surface [10,11,12]. Meanwhile, Si can influence the precipitates including M_23_C_6_, MX, and Laves phase, and eventually affect the mechanical properties. Kim et al., reported that an excessive amount of Si addition will increase the high-temperature δ-ferrite and Laves precipitation phase and therefore degrade the mechanical properties [13,14]. In contrast, Hurst et al. found that Si has little effect on the formation of carbide phases [15].

Up to now, a lot of studies have been performed on 9Cr-F/M steels, including the influence of different normalizing and tempering processes on the mechanical properties [16], microstructure evolution at high temperatures [17,18], creep behavior under various aging conditions [19,20], the effect of alloying elements [13,21,22], and many other properties [23,24,25]. Nevertheless, limited studies on the microstructure changes of the sample surface and cross section under different stress states of 9Cr-F/M steel were carried out. Besides, for the safety performance of cladding material in service, it is necessary to evaluate the failure mechanism of 9Cr-F/M steels at different temperatures. Therefore, in this work, silicon contained 9Cr-F/M steel at different temperatures was systematically investigated by using a tensile tester and electron backscatter diffraction (EBSD).

## 2. Experimental

### 2.1. Material Preparation

**Figure 1:**
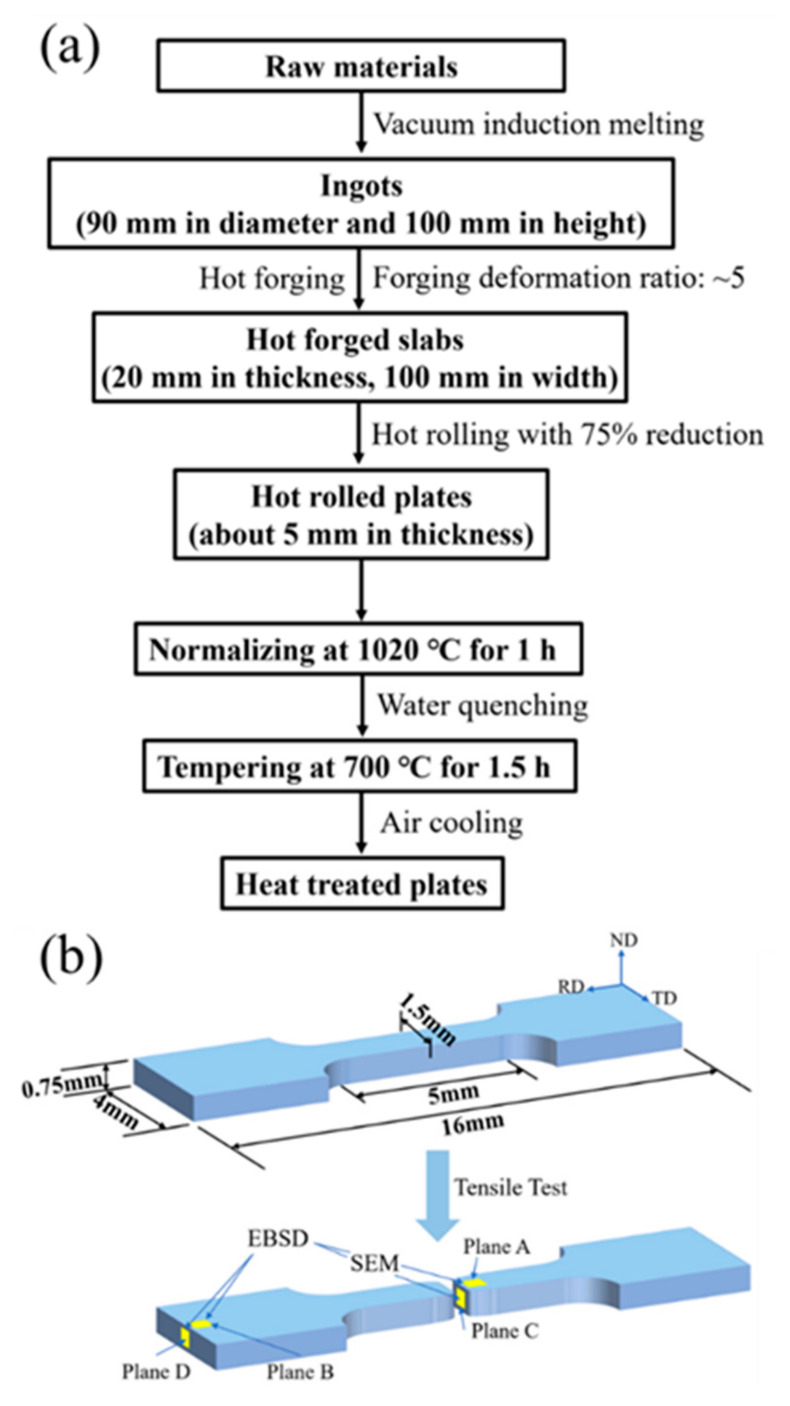
A schematic diagram of (**a**) manufacturing and heat treatment process and (**b**) microstructure characterization zone.

A schematic diagram of the manufacturing and heat treatment process is given in Figure 1a. 9Cr-F/M steel was fabricated by vacuum induction melting technique with nominal composition listed in Table 1. The ingots were hot-forged, hot-rolled, normalized at 1020 °C for 60 min followed by water quenching, and then tempered at 700 °C for 90 min followed by air cooling, sequentially. It is necessary to point out that after the hot rolling process, the rolling direction, transverse direction, and normal direction are referred to as RD, TD, and ND for short, respectively.

### 2.2. Mechanical Testing

For tensile tests, the specimens were cut into dog-bone-shaped specimens with a rectangular cross-section of 1.5 × 0.75 mm^2^ and a total length of 16 mm (Figure 1b), and then mechanically polished to remove the cutting-induced scratches. The tensile specimens were tested along the RD direction using an Instron-5967 machine with a constant speed of 0.3 mm/min in ambient air at room temperature (RT), 400 °C, and 550 °C, respectively. To ensure the uniformity of sample temperature during the tensile test, the sample was kept at the test temperature for 20 min before each test. For accuracy, each test was repeated at least three times. After the tensile test, the Vickers micro-hardness was performed in the non-deformed zone far away from the fracture with a load of 200 g and a dwell time of 15 s.

### 2.3. Microstructure Characterization

The sample after mechanical testing is used for microstructure characterization, the characterization area is shown in the yellow filled box in Figure 1b. Before characterization, specimens were placed flat together and fixed by the hot mounting press with metallographic mounting powder, followed by sanding with 400, 800, 1500, 2000 mesh SiC sandpapers, then mechanically polished to a mirror surface with a polishing cloth, and finally subjected to vibration polishing using an oxide polishing suspension for several hours to reduce surface stress, and thus, to achieve a high-quality surface. Microstructure features of specimens were characterized using scanning electron microscopy (SEM, Sirion 200, FEI, Portland, OR, USA) operated at 10 kV and Electron Backscatter Diffraction Pattern (EBSD, Oxford Instruments, Oxford, UK) operated at 15 kV. EBSD data were analyzed using HKL-Channel 5 software. To distinguish low-angle grain boundaries (LAGBs) and high-angle grain boundaries (HAGBs), the misorientation angle was set as >2° with red lines and >15° with black lines [26], respectively.

To investigate the heat-treated microstructure including the size and distribution of second phase particles, a transmission electron microscope (TEM, Tecnai G2 F20) was used. The TEM foils were prepared by mechanically polishing, electrochemical polishing using a Struers Tenupol-5 twinjet electro-polisher, and ion thinning using a Gatan model 691 precision ion-milling machine operated at 3.0 kV and 3° for 3 h, subsequently.

## 3. Results

**Figure 2:**
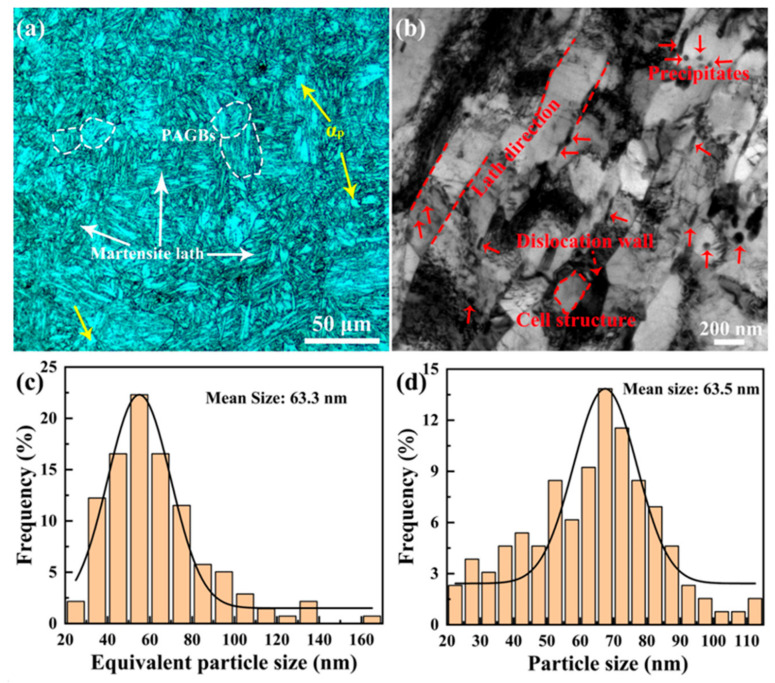
(**a**) Metallographic and (**b**) TEM image of the 9Cr-F/M steel, (**c**) frequency histogram of equivalent particle size of M_23_C_6_ phase and (**d**) frequency histogram of particle size of MX phase.

Figure 2 shows metallographic and TEM images, and histograms of the size distribution of M_23_C_6_ and MX phases of the 9Cr-F/M steel. Prior austenite grain boundaries (PAGBs), polygonal ferrite (αp) and martensite appear in the microstructure of the steel, as marked in Figure 2a. Martensitic laths with high dislocation density are discernible in Figure 2b, a great number of precipitations nucleated on the lath boundaries or within the sub-grains [17]. The precipitates mainly exist in two forms: one is relatively large and can be regarded as Cr-riched M_23_C_6_ [27,28]. Statistical analyses of 139 such precipitations indicated that they have a length (L) in the range of ~47–239 nm and width (W) in the scope of ~13–118 nm. Correspondingly, the average equivalent size described by √LW [29] is ~63.3 nm, as displayed in Figure 2c. The other small size can be thought as MX (M = V/Ta/Si, X = C, N) [28,30,31] from the EDS maps (not shown here). A total of 130 such phases were analyzed and the results suggested a size distribution of ~21–144 nm, with a mean size of 63.5 nm, as shown in Figure 2d.

### 3.1. Mechanical Properties

**Figure 3:**
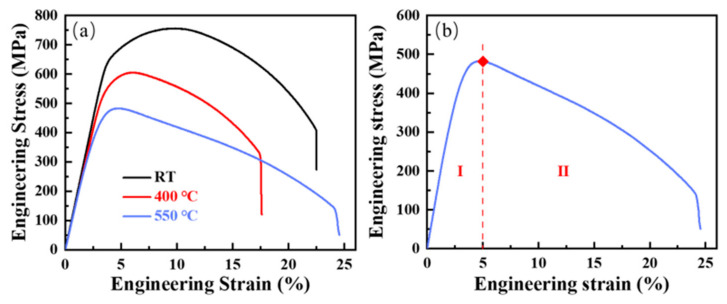
Engineering stress–strain curve of (**a**) 9Cr-F/M steel at constant RT, 400 °C and 550 °C, and (**b**) engineering stress–strain curve with two stages of 9Cr-F/M steel at 550 °C.

Typical engineering stress–strain curves (Figure 3a) of 9Cr-F/M steel were drawn using the data obtained by tensile testings under different conditions. It can be seen that temperature affects flow stress [32]. Under the constant loading rate selected in the test, when the temperature increases, the flow stress decreases. There is no obvious transition from the yield limit to the tensile limit (i.e., the peak stress) in the tensile curves of the steel at different temperatures, suggesting that this steel has a continuous yield and appreciable formability [33]. In addition, the engineering stress–strain curve can be divided into two stages (Figure 3b) according to its shape. In the first region (Stage I), uniform deformation prevails. When the stress exceeds the elastic limit, plastic deformation begins and deformation strengthening occurs simultaneously, making the stress increase with increasing strain until peak stress is reached. In the second region (Stage II), after the stress reaches the tensile limit, necking forms and the stress decreases with increasing strain until it fractures completely.

**Figure 4:**
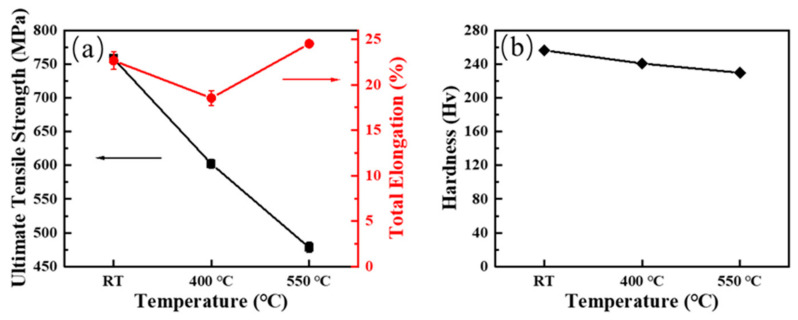
Variation of (**a**) ultimate tensile strength and total elongation, and (**b**) hardness with temperatures of 9Cr-F/M steel.

Variation of the ultimate tensile strength (UTS), total elongation (TE), and hardness with temperatures of 9Cr-F/M steel were displayed in Figure 4. It can be seen that with temperature increasing from RT to 550 °C, the UTS and hardness of the steel show a decreasing trend overall, although the UTS decreases more, while the hardness only slightly decreases. Whereas, the total elongations of the steel are V-shaped with temperature changes. The TE of this steel at 400 °C is smaller than that at RT, and at 550 °C, the TE of this steel increases to be slightly higher than RT. This phenomenon also occurred in other ferritic-martensite steels [34,35,36]. This phenomenon may be related to dynamic strain aging (DSA) [37], which is caused by the interaction between solute atoms and dislocations, i.e., the formation of Cottrell atmosphere. Thereby, the strength is enhanced and the elongation is decreased. In addition, there may also exist a critical temperature [38], when the temperature exceeds this critical temperature, the obstructive effect of Cottrell atmosphere on the dislocation is weakened, thus plastic deformation is maintained, making the elongation increase.

### 3.2. Effect of Tensile Deformation on Microstructure

For convenience, the RD-TD plane near the fracture of the sample is marked as A, and the RD-TD section of the sample away from the fracture is marked as B. The ND-TD section where the sample fracture is located is marked as C, and the ND-TD section where the sample is far away from the fracture is marked as D, as shown in Figure 1b. Therefore, planes A and C are in the necking zone, planes B and D are in the non-deformation zone.

**Figure 5:**
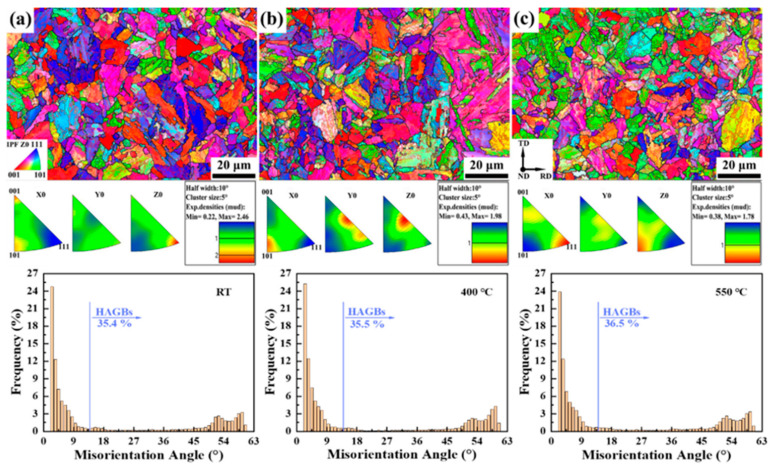
IPFs of plane B at (**a**) RT, (**b**) 400 °C, and (**c**) 550 °C of 9Cr-F/M steel and the histogram of misorientation angle of Z0 direction corresponding to each above.

Figure 5 displays the IPFs maps with corresponding frequency distribution histogram of misorientation angle at three different temperatures of plane B of 9Cr-F/M steel. From Figure 5, we can see the existence of prior-austenite grain boundaries (PAGBs) and martensite lath, and there are many substructures distributed in the PAGBs. The IPFs indicate that after heat treatment, the rolling texture almost disappears. The histogram shows that the number of HAGBs and LAGBs of plane B (non-deformed zone) remains almost constant at RT and 400 °C, and their number changes only slightly when the temperature is elevated to 550 °C. This is because 400 °C is in the range of low-temperature recovery (0.1–0.3 Tm, about 153–459 °C for this steel), in which the movement of vacancies mainly occurs and dislocation movement is not stimulated. At 550 °C, it is in the medium recovery stage (0.3–0.5 Tm, about 459–765 °C for this steel). In this stage, in addition to the movement of vacancies, the dislocations also begin to move. However, due to the short holding time, the number of dislocations is not significantly reduced, so it has little effect on the microstructure.

**Figure 6:**
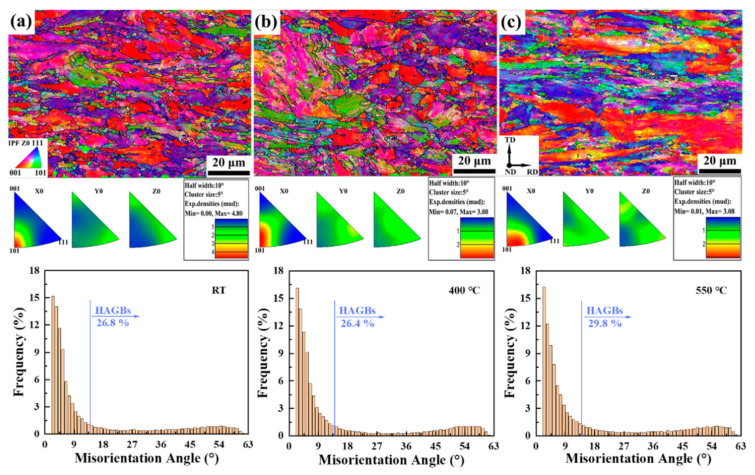
IPFs maps of plane A at (**a**) RT, (**b**) 400 °C, and (**c**) 550 °C of 9Cr-F/M steel and the histogram of misorientation angle of Z0 direction corresponding to each above.

Compared with plane B, plane A was continuously subjected to tensile stress during the stretching process due to being close to the fracture, so the grains were elongated to the direction of tensile stress, and a little texture was produced in the <101> direction, yet it was still not obvious, as shown in Figure 6. In addition, it can be found that after tensile deformation, the number of HAGBs is significantly reduced and the number of LAGBs is significantly increased no matter what the temperature is. Furthermore, just like plane B, there is little difference in the number of HAGBs and LAGBs at three chosen temperatures. The boundary of the substructure formed by rolling is the lattice distortion zone, with a large number of dislocations piled up, while the lattice inside the substructure is relatively complete, this substructure is called a cellular substructure [32], as marked in Figure 2. Dislocations are mainly concentrated in the cell wall, and there is only a sparse dislocation network within the cell. Stretching deformation will increase the number of cells and decrease their size, and the misorientation between the cells gradually increases, and their shape changes with the change of the grain shape. Although there is short-time low-temperature and medium-temperature recovery before stretching at 400 and 550 °C, its effect on reducing the number of dislocations is very limited.

**Figure 7:**
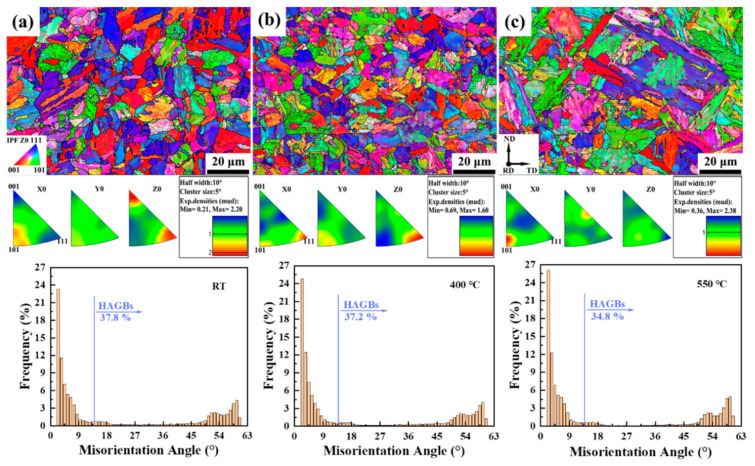
IPFs maps of plane D at (**a**) RT, (**b**) 400 °C, and (**c**) 550 °C of 9Cr-F/M steel and the histogram of misorientation angle of Z0 direction corresponding to each above.

IPFs maps of plane D at RT, 400 °C, and 550 °C of 9Cr-F/M steel and the histogram of misorientation angle corresponding to each above are shown in Figure 7. Plane D is similar to plane B, which is also a non-deformation zone far away from the fracture, but is viewed from a different direction. However, it is strange that at 550 °C, the number of HAGBs is less than that at RT and 400 °C, which is different from the case of plane B. Careful observation revealed that this is related to the microstructure of the EBSD scan area. Specifically, the microstructure of plane D at 550 °C is mostly coarse martensite, and the number of PAGBs is small, which means that plane D at 550 °C will have more substructures. However, the microstructure of plane D at RT and 400 °C is mostly PAGBs, with less coarse martensite.

**Figure 8:**
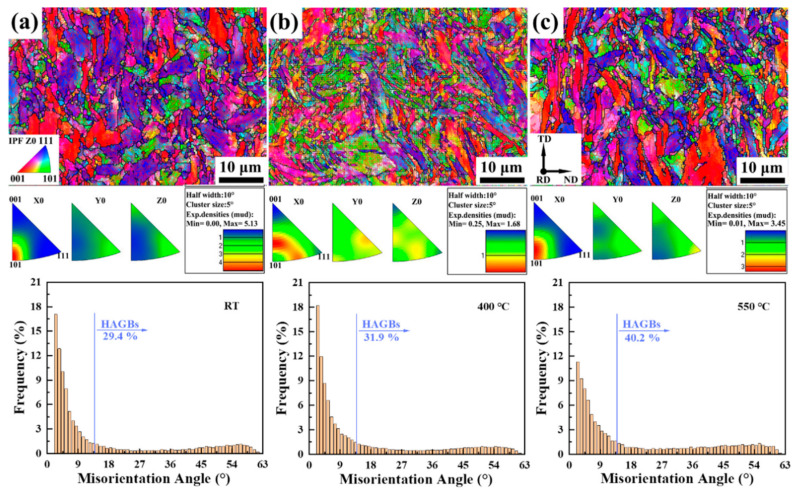
IPFs maps of plane C at (**a**) RT, (**b**) 400 °C, and (**c**) 550 °C of 9Cr-F/M steel and the histogram of misorientation angle of Z0 direction corresponding to each above.

Corresponding to plane D is plane C. Plane C is the place where the fracture is located, i.e., it is the cross-section of the necking zone. Figure 8 shows the orientation and grain boundary maps of the section (plane C) of the necking zone at different temperatures and the histogram of misorientation angle corresponding to each above. It can be observed that the grain size of the cross-section of the necking zone was reduced compared to the plane D and plane A, and was not equiaxed, but elongated to be approximately parallel to the TD direction, which corresponds to the long side of the working area section of the sample. The histogram results of the misorientation angle at the three temperatures suggest that as the temperature increases, the quantity of HAGBs rises, and the number of LAGBs decreases, which is different from that of plane B near the fracture. In the tensile process of steel, the grains were elongated under the action of tensile stress, and the cross-section of the grains becomes smaller. Viewed from the cross-section, it is equivalent to the cross-sectional grain refinement, thus HAGBs increase. At high temperatures, the material softens, and the greater the elongation, the more obvious the grain cross-section refinement effect.

**Figure 9:**
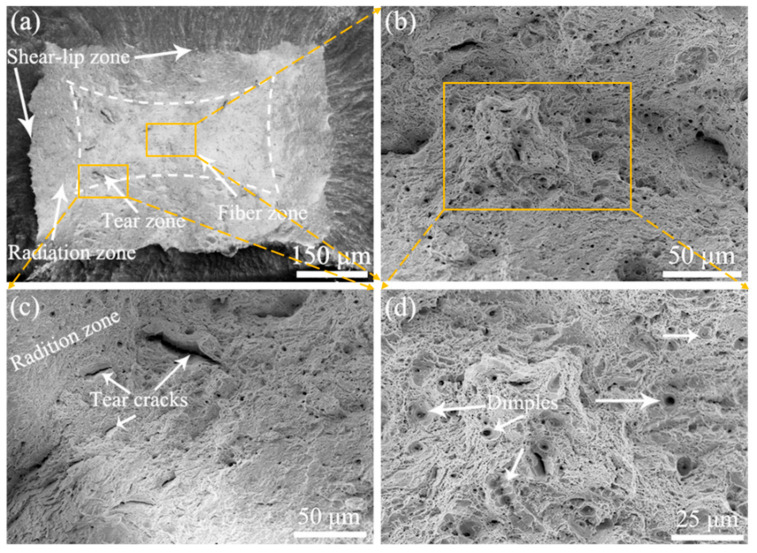
Fracture morphology of 9Cr-F/M steel at RT: (**a**) macro view presenting rectangular necking, (**b**) magnified image of the fiber zone, (**c**) magnified view of the radiation zone, and (**d**) magnified image from the middle of (**b**).

**Figure 10:**
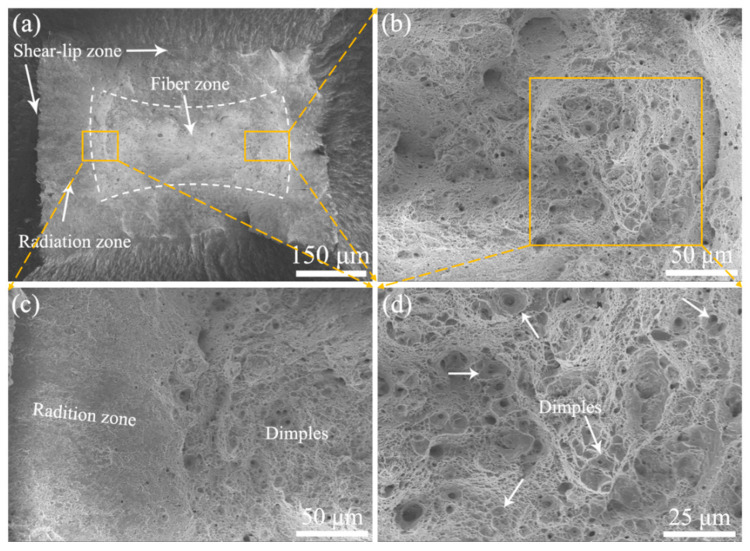
Fracture morphology of 9Cr-F/M steel at 400 °C: (**a**) macro view presenting rectangular necking, (**b**) magnified image of the fiber zone, (**c**) magnified view of the selected radiation zone, and (**d**) magnified image from the middle of (**b**).

**Figure 11:**
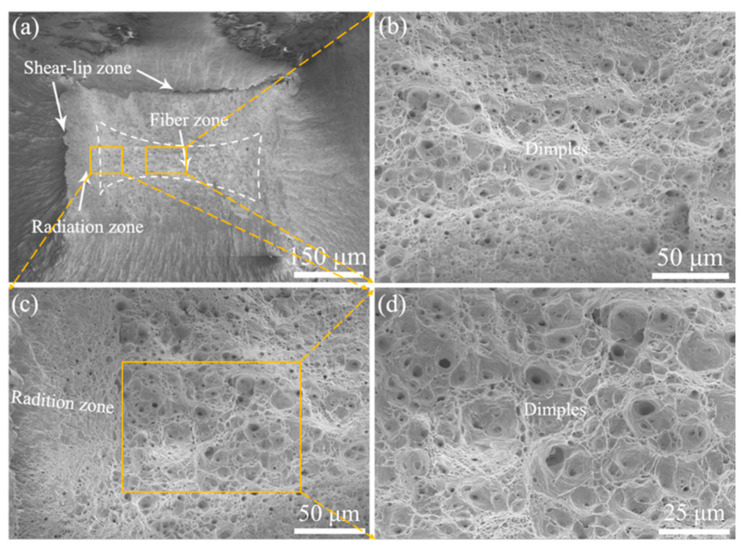
Fracture morphology of 9Cr-F/M steel at 550 °C: (**a**) macro view presenting rectangular necking features, (**b**) magnified image of the fiber zone, (**c**) magnified view of the selected radiation zone, and (**d**) magnified image from the middle of (**c**).

Figure 9, Figure 10 and Figure 11 shows the fracture morphology of 9Cr-F/M steel after stretching at three different temperatures, all of which reveal obvious necking features. Two modes of fracture are observed [13]. Compared with the rod-shaped tensile sample, since the working area of the tensile sample in our experiment is rectangular, the fracture is not a typical cup-cone shape [13,32,33], but it still has the macroscopic characteristics of typical plastic fracture (the shear-lip zone at the outside, the fiber zone at the center and the radiation zone between them), as shown in Figure 9, Figure 10 and Figure 11a. Besides, at RT, ductile dimples of the fiber zone and tear cracks of the tear zone were mixed, but at 400 and 550 °C, only the fiber zone exists, as drawn with white dashed lines. According to the shape of the dashed area, the deformation in the TD direction of the sample was more severe than that in the ND direction, which suggested that during necking, the force in the TD direction on the cross-section was greater than that in the ND direction, so that the grain was elongated in the TD direction, which is in agreement with the grain being elongated to the TD direction in Figure 8. Moreover, as the temperature increases, the size of the dimples also increases, although the order of TE is 550 °C > RT > 400 °C. This means that there is a deeper reason that brings about this phenomenon. In-situ high-energy X-ray diffraction and small-angle X-ray scattering were performed by Leyun Wang et al. [34] to characterize this phenomenon, and the results showed that the decrease of elongation at 400 °C may be related to the load transfer between the matrix and the precipitated phases during the stretching process.

## 4. Conclusions

In the present study, high-temperature tensile testing and microstructural characterization of the stretched samples of 9Cr-F/M steel were carried out. The main results can be concluded as follows:

(1) The steel consists of a typical martensitic structure with only a few polygonal ferrites embedded, and two kinds of precipitates nucleated on the lath boundaries or within the sub-grains. One is the M_23_C_6_ phase, the other is the MX phase.
(2) The UTS for the steel is in the order of RT > 400 °C > 550 °C, but the TE is in the sequence of 550 °C > RT > 400 °C.
(3) For the RD-TD plane in the necking zone, tensile deformation made the grain elongated to the direction of applied stress and increased the quantity of LAGBs. In the cross-section (ND-TD plane) of the necking zone, equivalent grain refinement occurs within the grain cross-section due to the elongation of the grains.
(4) For samples with rectangular working area cross-section, the deformation in the TD was more severe than that in the ND, which made the grain to be elongated to the TD direction.

## Figures and Tables

**Table 1:** Chemical composition (wt.%) of the 9Cr-F/M steel.

Designation	Fe	Cr	C	W	Si	Mn	Ta	V	Zr
9Cr-F/M	Bal.	9	0.12	1.5	0.7	0.5	0.1	0.2	0.01

## Data Availability

Not applicable.
